# Novel approaches for separating graft-versus-leukemia effects from graft-versus-host disease

**DOI:** 10.3389/fonc.2026.1838922

**Published:** 2026-05-29

**Authors:** Yuta Hasegawa, Takanori Teshima, Daigo Hashimoto

**Affiliations:** Department of Hematology, Hokkaido University Faculty of Medicine, Sapporo, Japan

**Keywords:** graft-versus-host disease, graft-versus-leukemia effects, immune evasion, tissue tolerance, tumor immune microenvironment

## Abstract

Allogeneic hematopoietic cell transplantation (allo-HCT) is a curative therapy for various hematologic malignancies. Separation of graft-versus-leukemia (GVL) effects from graft-versus-host disease (GVHD) remains one of the central challenges in allo-HCT. However, relapse after allo-HCT remains associated with poor prognosis, and effective strategies that preserve graft-versus-leukemia (GVL) effects while preventing graft-versus-host disease (GVHD) are still lacking. Recent advances have revealed that post-transplant relapse is driven by diverse mechanisms, including leukemia-intrinsic immune escape, donor T-cell exhaustion, and persistence of leukemia stem cells. Emerging therapeutic strategies, such as selective modulation of T-cell trafficking and enhancement of tissue tolerance, are promising approaches to ameliorate GVHD without attenuating GVL effects. Additional approaches including cellular therapies, selective immune modulation, and restoration of leukemia immunogenicity are also being actively explored. Furthermore, restoration of leukemia immunogenicity and targeted elimination of leukemia stem cells are expected to provide new opportunities to prevent relapse without aggravating GVHD. In addition, approaches that reshape the tumor immune microenvironment or modulate T-cell exhaustion may further strengthen GVL activity while limiting harmful alloreactivity. These developments suggest that the long-considered difficult goal of separating GVHD from GVL effects may become achievable in the near future. In this review, we provide an overview of novel therapeutic targets aimed at achieving the separation of GVHD and GVL, with a particular focus on findings related to acute myeloid leukemia.

## 1 Introduction

Allogeneic hematopoietic stem cell transplantation (allo-HCT) is a curative treatment for various hematologic malignancies. However, donor T cells, the major mediators of graft-versus-leukemia (GVL) effects, can simultaneously cause a life-threatening complication, graft-versus-host disease (GVHD). This therapeutic dilemma arises because many alloantigens, including MHC molecules and minor histocompatibility antigens, are shared between leukemia cells and healthy recipient tissues, making selective targeting inherently difficult (1, 2). The separation of these two effects is crucial for improving transplant outcomes and has remained a central research challenge in the field of transplantation. In the post-transplant setting, where a donor-derived immune system is established, leukemia relapse requires escape from donor-derived GVL effects through specific mechanisms. In recent years, advances in large-scale registry analyses and comprehensive immune profiling techniques, such as single-cell RNA sequencing, have markedly accelerated research into the mechanisms of post-transplant relapse as well as the pathophysiology of GVHD, leading to substantial new insights. In this review, we provide an overview of novel therapeutic targets aimed at achieving the separation of GVHD and GVL, with a particular focus on findings related to acute myeloid leukemia (AML). Relapse after allo-HCT is associated with dismal survival, and no universally effective standardized treatment strategy currently exists.

## 2 Mechanisms of immune evasion by leukemia cells after allo-HCT

The mechanisms underlying AML relapse can be broadly categorized into leukemia-intrinsic mechanisms and those derived from immune cells or the immune microenvironment. Leukemia-intrinsic mechanisms include loss of the mismatched HLA haplotype due to loss of heterozygosity of the short arm of chromosome 6 (6p-LOH), where HLA genes are located, downregulation of HLA class II expression through epigenetic alterations, suppression of immune responses via expression of inhibitory molecules such as PD-L1, and enhanced proliferative capacity driven by clonal evolution of leukemia cells. In contrast, immune-derived mechanisms include functional impairment due to exhaustion of alloreactive donor T cells, the presence of an immune-cold tumor microenvironment, and escape of leukemia cells to extramedullary sites outside the bone marrow. Unlike many solid tumors, AML generally carries a relatively low mutational burden and limited neoantigen repertoire, suggesting that post-transplant immune escape may depend less on neoantigen loss and more on impaired antigen presentation, low baseline immunogenicity, and immune suppressive microenvironments (3).

### 2.1 HLA loss and HLA mutation in leukemia cells

In pathological conditions such as aplastic anemia, where hematopoietic cells are targeted by T cells, immune escape through 6p loss of heterozygosity (6p-LOH) affecting HLA loci has been reported (4). AML cells, as malignant hematopoietic cells, are presumed to be subjected to similar immune pressure. Masuda et al. reported that HLA loss due to 6p-LOH increases at the time of leukemia relapse, including post-transplant relapse (5). Furthermore, their study demonstrated cases in which HLA expression was restored by IFN-γ stimulation, suggesting that both complete HLA loss and downregulation of HLA expression contribute to immune evasion by AML. Direct evidence that 6p-LOH contributes to post-transplant relapse was provided by Vago and colleagues through detailed chimerism analyses of post-transplant cases (6). In their study, chimerism following HLA-haploidentical transplantation was evaluated using both short tandem repeat (STR) analysis, which detects donor and recipient genetic contributions based on polymorphic DNA repeat markers, and HLA haplotype analysis. In some AML relapse samples, STR analysis detected recipient-derived cells, whereas HLA analysis identified only donor-type HLA. This discrepancy was shown to result from loss of the recipient-derived HLA haplotype caused by 6p-LOH. Such HLA loss has been reported to occur at high frequency after HLA-mismatched transplantation, including haploidentical transplantation (7).

From the perspective of identifying genetic abnormalities specific to post-transplant relapse, several studies have investigated whether distinct driver mutations characterize these cases. However, these analyses have not identified specific driver mutations uniquely associated with post-transplant relapse. Rather, they have consistently shown an increased frequency of HLA-related mutations after transplantation (8–10). In contrast, such enrichment of HLA alterations is not observed in relapse following chemotherapy alone, further suggesting that post-transplant relapse is driven, at least in part, by immune escape mechanisms mediated not only by 6p-LOH but also by various HLA mutations (10). Importantly, relapse mediated by HLA haplotype loss would not respond to donor lymphocyte infusion or a second transplant from the same donor, highlighting the clinical importance of identifying this mechanism (11).

### 2.2 HLA class II downregulation

HLA loss is frequent in haploidentical transplantation, but rare in HLA-matched transplantation (9, 12). Christopher and colleagues performed integrated analyses combining exome, transcriptome, epigenome, and flow cytometry of leukemic cells from patients who experienced AML relapse after transplantation or chemotherapy. They reported that, in relapse after HLA-matched transplantation, epigenetic alterations such as methylation of the CIITA gene lead to reduced MHC class II expression (HLA silencing) at a high frequency (9). Similar findings were also supported by subsequent immune profiling studies of post-transplant AML relapse, which demonstrated recurrent downregulation of HLA class II and distinct immune escape signatures in relapsed AML after allo-HCT (13). Such HLA class II silencing can be readily detected by flow cytometry, and HLA class II expression can be restored by inflammatory stimuli such as IFN-γ. (13).

### 2.3 T-cell suppression by leukemia cells

Although HLA loss or silencing represents an important mechanism of immune evasion in leukemia, relapse frequently occurs in the absence of these mechanisms. AML cells at post-transplant relapse have been reported to exhibit increased expression of inhibitory molecules such as PD-L1 and B7-H3. Interestingly, a positive correlation has been observed between HLA expression levels and PD-L1 expression levels. In post-transplant relapse of AML with high HLA expression, PD-L1 expression tends to be relatively high, whereas in AML with reduced HLA expression, PD-L1 expression tends to be lower. These findings suggest that, in AML without downregulation of HLA expression, immune evasion may be achieved through inhibitory receptors that suppress T-cell responses (13).

### 2.4 Donor T-cell exhaustion

After allo-HCT, alloantigens expressed on recipient non-hematopoietic cells persist throughout life and continuously stimulate the corresponding alloreactive donor T cells. Using a murine model, we and others demonstrated that donor T cells differentiate into exhausted T cells after allo-HCT, leading to leukemia relapse (14, 15). Analyses of clinical samples have further shown that, compared with T cells at leukemia diagnosis or during post-transplant remission, T cells at post-transplant relapse exhibit increased PD-1 expression, suggesting progression of T-cell exhaustion (13).

These data suggest that T-cell exhaustion contributes to leukemia relapse. However, it should also be considered that leukemia proliferation itself may increase alloantigen burden and thereby induce T-cell exhaustion. Therefore, it remains unclear whether T-cell exhaustion is a cause or a consequence of leukemia relapse. Noviello et al. analyzed bone marrow CD8^+^ T cells after HLA-matched allogeneic transplantation and demonstrated that exhaustion markers such as PD-1 and TIM-3 are upregulated at relapse. Moreover, they showed that terminal exhaustion of bone marrow stem cell memory CD8^+^ T cells at approximately two months post-transplant predicts subsequent relapse, suggesting a causal role of T-cell exhaustion in leukemia relapse (16). The establishment of more simplified approaches to evaluate T-cell wdysfunction may aid in predicting post-transplant relapse and guiding early therapeutic intervention. In addition, recent studies suggest that post-transplant T-cell dysfunction is likely multifactorial and may also be shaped by pharmacologic immunosuppression, microbial dysbiosis, and environmental cues within the host microenvironment (17).

### 2.5 Residual leukemia stem cells

LSCs are characterized by proliferative restriction, quiescence, and self-renewal capacity, and they highly express BCL-2 family molecules. Due to their quiescent state and high BCL-2 expression, LSCs tend to persist after chemotherapy and contribute to measurable residual disease (MRD) (18, 19). In addition to chemoresistance, LSCs have also been shown to possess immune-evasive properties, suggesting their potential involvement in post-transplant relapse (20). TIM-3 is highly expressed in LSCs in the majority of AML cases regardless of mutation or subtype. In contrast, it is minimally expressed in normal hematopoietic stem cells and remains persistently expressed in LSCs at relapse. Therefore, TIM-3 is expected to serve as a potential MRD marker after transplantation (21). In the bone marrow stem cell fraction (CD34^+^CD38^-^) of patients early after allogeneic transplantation for high-risk AML, cases in which the proportion of TIM-3-positive cells (TIM-3^+^ LSCs) was less than 60% had a relapse rate of 22.2%, whereas 87.5% of cases with ≥ 60% TIM-3^+^ cells experienced relapse. These findings suggest that residual LSCs after transplantation represent a therapeutic target for preventing post-transplant relapse (22).

### 2.6 Tumor immune microenvironment

Vadakekolathu et al. performed a comprehensive mRNA analysis of the TiME in AML and calculated an immune-infiltrated score based on the expression of genes related to interferon function, adaptive immune responses, and myeloid cells. AML cases were then classified into two groups: immune-infiltrated AML with a high immune-infiltrated score and immune-depleted AML with a low score. The prognostic impact of these distinct tumor microenvironment subtypes differed markedly according to the European LeukemiaNet (ELN) 2017 genetic risk stratification system, which categorizes AML into favorable, intermediate, and adverse risk groups based on cytogenetic and molecular abnormalities (23). In the ELN2017 favorable group, immune-infiltrated AML showed significantly superior relapse-free survival (RFS) and overall survival (OS) compared with immune-depleted AML. In contrast, in the ELN2017 adverse group, immune-depleted AML was associated with significantly better outcomes. In the ELN2017 intermediate group, no significant difference in prognosis was observed between the two subtypes. From the opposite perspective, these findings indicate that ELN2017 risk stratification effectively discriminates prognosis in immune-infiltrated AML, whereas it fails to stratify outcomes in immune-depleted AML. A representative example of immune-infiltrated AML within the ELN2017 adverse group is TP53-mutated AML. Although such cases exhibit marked inflammatory cell infiltration, they are characterized by T-cell dysfunction and abundant infiltration of immunosuppressive cells, including regulatory T cells and myeloid-derived suppressor cells (MDSCs) (24, 25). In contrast, the TiME in FLT3-ITD-positive AML is thought to be characterized by less infiltration of T cells and NK cells, resulting in a relatively weak immune response. Such a TiME may be modifiable by FLT3 inhibitors, as will be discussed below (24, 26). Importantly, patients with immune-infiltrated AML categorized in the ELN2017 adverse risk group derived the greatest benefit from allo-HCT, suggesting that infiltration of donor-derived immune cells may establish a new TiME that is unfavorable for AML persistence. Collectively, these observations highlight the importance of the TiME in regulating AML prognosis, suggesting that the TiME is a promising target for preventing post-HCT relapse without exacerbating GVHD.

### 2.7 Extramedullary relapse

Because extramedullary relapse of AML is frequently observed after allo-HCT, it has been suggested that extramedullary disease may represent a form of immune evasion (27). A recent report analyzing skin relapse cases using single-cell RNA sequencing demonstrated abundant infiltration of T cells, B cells, and macrophages in cutaneous AML lesions (leukemia cutis), indicating an immune-infiltrated type of TiME (28). In post-transplant analyses, changes in T-cell chimerism were slower in the skin, and recipient-derived T cells were more likely to persist. Compared with bone marrow relapse, extramedullary relapse was associated with reduced HLA II expression, suggesting a potential mechanism of immune evasion. In addition, extramedullary relapse lesions in the skin showed abundant infiltration of T cells expressing high levels of exhaustion-associated molecules such as PDCD-1, LAG3, and CTLA-4. These findings suggest that, in a subset of extramedullary relapse cases, despite prominent inflammatory cell infiltration, donor T-cell exhaustion within the TiME and reduced HLA II expression on leukemia cells may facilitate immune escape more readily than in bone marrow relapse. In contrast, some extramedullary lesions may occur in sanctuary sites with immune privilege, thereby avoiding infiltration by immune-competent cells, such as in the central nervous system. Although further investigation is required, a detailed understanding of the mechanisms underlying extramedullary relapse is likely to be crucial for preventing post-transplant relapse.

## 3 Prevention of post–allo-HCT relapse without exacerbating GVHD

As strategies to separate graft-versus-leukemia (GVL) effects from graft-versus-host disease (GVHD), two major approaches can be considered. One approach focuses on refining GVHD prevention and treatment to avoid compromising GVL effects. The other aims to maximize GVL effects without exacerbating GVHD. Ideally, it would be possible to enhance GVL effects while simultaneously improving GVHD prophylaxis.

### 3.1 Development of GVHD prevention and treatment strategies that do not compromise GVL effects

#### 3.1.1 Intensification of local therapy

In the treatment of mild skin and gastrointestinal GVHD, topical corticosteroids have been used without attenuating GVL effects, however, they are insufficient for severe GVHD. Strengthening local GVHD therapy may represent a more direct approach to separating GVL from GVHD. We recently demonstrated that topical ruxolitinib effectively ameliorates skin GVHD in mice, whereas topical corticosteroids exert direct toxicity on skin stem cells (29). Long-term use of corticosteroids is associated with adverse events such as skin atrophy and delayed wound healing. In contrast, ruxolitinib protects skin stem cells from GVHD and promotes wound healing (29). Currently, the development of a ruxolitinib cream formulation for acute and chronic cutaneous GVHD is underway (30). Markova and colleagues conducted a placebo-controlled study in patients with steroid- and topical calcineurin inhibitor-refractory chronic cutaneous GVHD, applying ruxolitinib cream to one area and placebo to another. They demonstrated a significant reduction of GVHD only in the ruxolitinib-treated areas (31). Topical approaches may reduce systemic immunosuppression; however, careful evaluation of local irritation, delayed wound healing, infection risk, and long-term tolerability remains necessary.

#### 3.1.2 Inhibition of donor T-cell trafficking to GVHD target organs

Although recipient-derived alloantigens are expressed in all tissues, the primary target organs of acute GVHD are the skin, gastrointestinal tract, and liver, suggesting that donor T cells preferentially migrate to these organs. Therefore, selective modulation of tissue-specific T-cell trafficking pathways, without inhibiting migration to the bone marrow, has emerged as a promising strategy to suppress GVHD while preserving GVL effects.

In intestinal homing, the interaction between α4β7 integrin expressed on T cells and MAdCAM-1 expressed in the tissue has been shown to be essential in gut GVHD (32). High expression of MAdCAM-1 has been observed on vascular endothelial cells surrounding the crypt base of the intestine, and in murine GVHD models, donor T cells migrate to the crypt base as early as day 4 after transplantation. Blocking the MAdCAM-1-α4β7 integrin interaction has been shown to protect intestinal stem cells residing within the crypts (33). Vedolizumab, a humanized monoclonal antibody targeting α4β7 integrin, reduced lower gastrointestinal acute GVHD compared with placebo in a randomized phase III trial (34). Potential limitations of α4β7 blockade include increased susceptibility to enteric infections, impaired intestinal immune surveillance, and delayed immune reconstitution (35).

Sphingosine-1-phosphate (S1P) is a metabolic product of sphingolipids, which are structural components of biological membranes. S1P regulates the trafficking of various immune cells through interaction with S1P receptors (S1PR1-5), with S1PR1 playing a particularly important role in T cells (36). Upon TCR and/or inflammatory stimulation, T cells transiently downregulate S1PR1 expression, rendering them insensitive to the high S1P concentrations in lymphatic fluid and thereby promoting their retention within lymph nodes, where they can receive more efficient antigen stimulation (37). After sufficient activation, S1PR1 expression is restored within a few days, enabling T cells to migrate into efferent lymphatics.

S1PR1 modulators such as mocravimod (KRP203) and the pan-S1PR modulator fingolimod (FTY720) have been shown in murine models to sequester donor T cells within lymph nodes, promote activation-induced cell death, and suppress migration to the GVHD target organs, thereby ameliorating GVHD (38, 39). Importantly, GVL effects were relatively preserved in S1PR modulator-treated mice (38, 40). In a phase I trial of post-transplant mocravimod administration, circulating lymphocyte counts decreased, while T-cell numbers in the bone marrow increased (41). These findings suggest that selective inhibition of the S1P-S1PR1 interaction can suppress donor T-cell trafficking to GVHD target organs while preserving migration to the bone marrow, thereby maintaining GVL effects. A randomized phase III trial evaluating post-transplant mocravimod administration, with leukemia-free survival in AML as the primary endpoint, is currently ongoing (MO-TRANS trial). Clinically relevant adverse effects of S1PR modulators may include bradycardia, hepatic dysfunction, macular edema, lymphopenia, and increased infection risk, which should be carefully balanced against their therapeutic benefit (4).

#### 3.1.3 GVHD prevention and treatment targeting tissue tolerance

Tissue tolerance is a recently proposed concept that is considered a mechanism for maintaining tissue homeostasis in the presence of immune responses, distinct from immune tolerance (42). In the post-transplant environment, where strong immune responses occur, acute GVHD may preferentially develop in tissues with lower tissue tolerance. Although further investigation is required to fully elucidate the underlying mechanisms, epithelial regeneration from tissue-resident stem cells clearly represents a tissue-intrinsic mechanism to preserve homeostasis and is considered a central component of tissue tolerance.

Tissue stem cells become targets of alloreactive T cells during GVHD, and impairment of their regenerative capacity contributes to persistent tissue damage. For example, intestinal stem cells located in the intestinal crypts differentiate into all epithelial cell lineages in the gut and maintain mucosal integrity. Therefore, injury to intestinal stem cells by alloreactive T cells leads to delayed repair of the mucosal epithelium (43–45). In other target organs of acute GVHD, including the skin and liver, LGR5-positive skin stem cells residing in hair follicles and colony-forming stem cells within the biliary epithelium, respectively, are targeted and damaged during acute GVHD, contributing to more severe tissue injury (29, 46). In the intestine, not only intestinal stem cells but also components of the intestinal stem cell niche are damaged. Lymphatic endothelial cells, which produce R-Spondin3, a factor essential for intestinal epithelial maintenance and proliferation, are injured during GVHD, resulting in reduced R-Spondin3 production in the intestine (47). In addition, IL-22 is a cytokine produced by type 3 innate lymphoid cells (ILC3) that exerts protective effects on intestinal stem cells. GVHD damages ILC3, leading to decreased IL-22 production, reduction of intestinal stem cells, and exacerbation of acute GVHD (44).

Enhancement of tissue tolerance has been shown to mitigate GVHD. Mechanisms of tissue stem cell injury vary among organs. In the intestine and skin, IFN-γ signaling contributes to stem cell injury, and the JAK2 inhibitor ruxolitinib has been shown to exert direct protective effects on stem cells (29, 48). In contrast, in the liver, TGF-β mediates injury to bile duct stem cells, and inhibition of TGF-β has been shown to protect bile duct stem cells in mouse models (46).

Furthermore, administration of growth factors for tissue stem cells has also been shown to suppress GVHD. Administration of intestinal epithelial growth factors such as R-Spondin1 and IL-22 protects intestinal stem cells and alleviates GVHD in mouse models, and recombinant IL-22 fused with immunoglobulin Fc is currently under clinical development (29, 44, 45, 49, 50). In addition, GLP-2, produced by intestinal L cells, functions as a growth factor for intestinal stem cells, and the GLP-2 agonist teduglutide is being developed as a therapeutic agent for acute GVHD (51–53). Because these approaches do not rely on immunosuppression, they can preserve GVL effects.

#### 3.1.4 GVHD treatment targeting inflammatory memory in epithelial stem cells

During GVHD treatment, re-exacerbation of disease, referred to as GVHD flare, frequently occurs, particularly during rapid tapering of corticosteroids. GVHD flare is more likely than initial GVHD to involve the gastrointestinal tract and to present with greater severity, making it a clinically significant challenge (54). Recently, a phenomenon in which tissue stem cells acquire epigenetic alterations following inflammation has been identified and is referred to as inflammatory memory. When tissue stem cells are exposed to inflammatory stimuli, chromatin accessibility increases through chromatin remodeling. Although many epigenomic changes are reversed after the resolution of inflammation, some persist, resulting in the retention of inflammatory memory. This inflammatory memory can promote more rapid activation of transcription factors upon subsequent inflammatory stimulation, potentially leading to more severe tissue injury or, conversely, enhanced tissue repair (55, 56).

Zhao et al. reported that GVHD induces metabolic reprogramming in ISCs, leading to succinate accumulation, which in turn alters DNA methylation and induces inflammatory memory in ISCs. As a consequence, these ISCs exhibit persistent defects in organoid formation and epithelial differentiation, reflecting a durable impairment in regenerative capacity that persists even after the resolution of GVHD (57, 58). We also demonstrated that epigenomic alterations occur in intestinal stem cells following GVHD (59). These changes were associated with enhanced MHC II expression and increased apoptosis in intestinal epithelial cells upon subsequent inflammatory stimulation. The prevention of inflammatory memory using epigenome-targeting agents may ameliorate GVHD without impairment of GVL effects. In addition, epigenome-targeting agents, including hypomethylating agents and inhibitors of HDAC, LSD1, EZH2 and other molecules are being actively developed as therapies for hematologic malignancies such as leukemia (60–62). Therefore, these agents may not only preserve GVL effects but also exert direct anti-leukemic activity. Targeting inflammatory memory through epigenetic or metabolic interventions may represent a novel strategy to prevent recurrent or steroid-refractory GVHD while preserving systemic antitumor immunity. Candidate approaches may include histone modification regulators, DNA methylation modulators, BET inhibition, and metabolic pathway targeting.

### 3.2 Strategies to enhance GVL effects without exacerbating GVHD

#### 3.2.1 Identification of minor histocompatibility antigens that selectively induce GVL effects

Minor histocompatibility antigens (MiHAs) are polymorphic peptides generated from genetic differences between donors and recipients and presented by shared HLA molecules (63–65). In contrast to mismatched major HLA antigens, MiHAs can differ even in HLA-identical donor-recipient pairs. Because the tissue distribution of MiHAs varies substantially, they represent an attractive framework for separating graft-versus-leukemia (GVL) effects from graft-versus-host disease (GVHD). Some MiHAs are broadly expressed in epithelial tissues and may contribute to GVHD, whereas others are selectively expressed in hematopoietic cells, including leukemia cells, and may preferentially mediate GVL effects (66–68). Donor T-cell responses directed against hematopoietic-restricted MiHAs may eliminate recipient leukemia cells and residual host hematopoiesis while sparing nonhematopoietic target organs such as the skin, liver, and gastrointestinal tract (69–71). This concept has long been regarded as one of the most biologically rational approaches for dissociating beneficial alloreactivity from harmful tissue injury after allo-HCT.

Cieri and colleagues analyzed whole-exome sequencing and transcriptomic data from 220 HLA-identical donor-recipient pairs undergoing allo-HCT for AML and MDS and identified MiHAs expressed in GVHD target organs that may contribute to GVHD development (72). Patients with a higher number of mismatches in these GVHD-associated MiHAs had a significantly increased risk of developing acute GVHD (grade II-IV). In this study, MiHAs selectively expressed in AML cells or hematopoietic cells were further extracted and were hypothesized to represent candidate GVL-inducing antigens. Among these GVL-associated antigens, those recurrently observed in patients who achieved GVHD-free, relapse-free survival (GRFS) were defined as GRFS-associated minor antigens. Patients harboring a greater number of these GRFS-associated minor antigens exhibited a lower relapse rate, indicating that these antigens contribute to the induction of GVL effects. Post-transplant tumor vaccination targeting these antigens may selectively enhance GVL effects without exacerbating GVHD. Future clinical trials are needed to validate this therapeutic strategy (72).

#### 3.2.2 Therapeutic strategies to suppress HLA silencing and enhance leukemia immunogenicity

As described above, epigenetic downregulation of HLA II expression contributes to immune evasion by leukemia cells and is implicated in post-transplant relapse (9). Because HLA II silencing can be readily reversed by inflammatory stimuli such as IFN-γ, relapse associated with HLA II silencing may be addressed by inducing IFN-γ production through interventions such as donor lymphocyte infusion (DLI), thereby restoring leukemia immunogenicity and preventing immune escape. However, such inflammation-inducing therapies may also exacerbate GVHD.

Alternatively, therapeutic strategies targeting HLA silencing in leukemia cells, which do not induce GVHD, are under active investigation. For example, inhibition of MDM2 has been reported to induce MHC I and MHC II expression in both murine and human AML cells. In addition, MDM2 inhibition increases the expression of tumor necrosis factor-related apoptosis-inducing ligand receptors 1 and 2 (TRAIL-R1/2) and enhances T cell-mediated cytotoxicity (73).

DNA methyltransferase inhibitors, such as azacitidine, have been shown to upregulate HLA expression. However, a randomized study failed to demonstrate a clinical benefit of azacitidine maintenance after allo-HCT (74). Notably, the same group subsequently published a retrospective study showing a favorable effect of azacitidine maintenance in the post-transplant setting (75). The authors speculated that the limited number of administered azacitidine cycles, largely due to logistical challenges, may have attenuated its therapeutic effect. Therefore, oral hypomethylating agents (CC-486) may offer a more feasible and effective approach for post-allo-HCT maintenance therapy, and the results of the ongoing randomized study are awaited (NCT04173533).

EZH2 inhibitors relieve repression of MHC II genes mediated by PRC2-dependent histone modifications. The use of these agents as post-transplant maintenance therapy may prevent immune escape and enhance GVL effects without inducing GVHD (76).

Menin inhibitors have demonstrated efficacy in relapsed or refractory AML harboring NPM1 mutations or KMT2A rearrangements by suppressing the expression of leukemogenic genes such as MEIS1 and HOXA (77). Recent studies have shown that MEIS1 suppresses the expression of CIITA and MHC II genes, and that menin inhibition activates CIITA and restores MHC II expression in AML cells (78). Interestingly, menin inhibition also induces moderate upregulation of MHC II expression in a subset of AML cases lacking NPM1 mutations or KMT2A rearrangements, suggesting that post-transplant maintenance therapy with menin inhibitors may have broad applicability. Furthermore, in AML with KMT2A rearrangements, menin inhibition promotes transcription of endogenous retroviruses (ERVs), which in turn activate the cGAS/STING pathway and contribute to increased MHC expression. These effects, including enhanced MHC expression and ERV activation, are thought to suppress immune escape of AML cells and promote GVL effects. Furthermore, the physiological menin-KMT2A complex induces the expression of genes that negatively regulate T cell activation, and menin inhibition enhances T cell effector function and cytokine production and inhibits T-cell exhaustion. Notably, in mouse models, menin inhibition has not been associated with exacerbation of GVHD (78).

#### 3.2.3 Interferon-alpha

During viral infection, IFN-α upregulates HLA I expression on infected cells, thereby enhancing antigen presentation and promoting their elimination by CD8^+^ T cells. In addition, IFN-α stimulates various immune cells and induces inflammatory responses, which likely indirectly promote HLA II expression. In the post-transplant setting, these effects may counteract HLA II silencing as described above, and upregulation of HLA I expression is expected to enhance CD8^+^ T cell-mediated elimination of leukemia cells.

Furthermore, IFN-α signaling has been shown to suppress GVHD. Using murine models, Robb et al. demonstrated that recipient deficiency of the IFN-α receptor (IFNAR) markedly exacerbates GVHD (79). Similarly, Fischer et al. reported that recipient deficiency of either cGAS/STING or RIG-I/MAVS, which are viral nucleic acid sensing pathways, also worsens GVHD (80). Because activation of these pathways promotes type I IFN production, these findings further support a protective role of IFN-α against GVHD. IFN-α directly acts on intestinal epithelial cells to enhance epithelial integrity and maintain mucosal barrier function, thereby limiting microbial translocation and attenuating intestinal inflammation, a central driver of GVHD pathogenesis (81).

In addition to its immunomodulatory effects, IFN-α exerts direct antitumor activity against myeloid malignancies. Although it has long been investigated as a post-transplant maintenance therapy, clinical use was limited by severe toxicities (82). More recently, pegylated IFN-α (peg-IFN), which has improved tolerability, has been evaluated in this setting. Magenau et al. conducted a phase I/II trial with peg-IFN every 14 days for a total of four doses starting one day prior to transplantation in high-risk AML patients and demonstrated that peg-IFN could be safely administered beginning prior to transplantation without exacerbating severe GVHD, possibly enhancing GVL effects (83). Similarly, Mo et al. reported that peg-IFN administration in AML patients with measurable residual disease (MRD) after transplantation resulted in high rates of MRD negativity (84).

More recently, ropeginterferon-α (Ropeg-IFN), a monopegylated IFN-α formulation with improved pharmacokinetics and safety, has been developed and is now a first-line therapy for polycythemia vera. Using murine transplantation models, we further demonstrated that Ropeg-IFN enhances antigen presentation by leukemia cells and, when administered at the time of transplantation, markedly enhances GVL effects while simultaneously suppressing GVHD (85). Furthermore, Ropeg-IFN exhibited partially selective antitumor activity against LSCs. These findings suggest that Ropeg-IFN represents a promising strategy for post-transplant maintenance therapy, and clinical trials evaluating its efficacy in preventing relapse while limiting GVHD are warranted.

#### 3.2.4 Therapeutic strategies targeting leukemia stem cells

LSCs exhibit strong resistance to chemotherapy and represent a critical therapeutic target for relapse prevention. Jeyaraju et al. investigated an *in vitro* model in which AML cells were exposed to low-dose azacitidine over an extended period and reported a reduction in the primitive LSC fraction (CD34^+^/CD38^-^) along with an increase in the more differentiated fraction (CD34^+^/CD38^+^). These changes were not associated with marked induction of apoptosis, suggesting that differentiation induction was the primary mechanism underlying LSC reduction. Furthermore, in a NOD/SCID mouse xenograft model using patient-derived AML cells, long-term oral azacitidine administration similarly reduced LSC frequency and promoted AML cell differentiation, which was associated with prolonged survival. Increased reactive oxygen species (ROS), mediated by induction of myeloperoxidase (MPO), was suggested as a potential mechanism driving LSC differentiation (86). These findings suggest that azacitidine may induce differentiation of chemotherapy-resistant LSCs into more mature phenotypes, and combination therapy with azacitidine and chemotherapy has attracted attention as a strategy to increase LSC sensitivity to treatment (19). In addition, post-transplant azacitidine administration has been shown to induce tumor-specific CD8^+^ T cell responses (87). In solid tumor models, combined exposure to IFN-γ and azacitidine has also been shown to markedly increase MHC I expression on tumor cells (88). These effects may help prevent immune escape of leukemia following transplantation.

Based on these mechanisms, including induction of LSC differentiation and enhancement of leukemia immunogenicity, clinical trials have evaluated hypomethylating agents (HMAs), such as azacitidine and decitabine, either as part of conditioning regimens or as post-transplant maintenance therapy to enhance GVL effects. Clinical trials incorporating decitabine into conditioning regimens demonstrated reduced relapse rates and improved overall survival, without increasing GVHD incidence (89, 90). However, azacitidine maintenance therapy requires outpatient administration for 5 to 7 days per cycle, which presents practical challenges. In a randomized trial evaluating post-transplant azacitidine maintenance, many patients were unable to complete therapy, with a median of only four cycles administered, and no significant improvement in relapse-free or overall survival was observed (74). Nevertheless, a retrospective analysis from the same group suggested that when maintenance therapy was adequately delivered, azacitidine maintenance was effective in high-risk AML/MDS patients, indicating that its therapeutic potential may depend on treatment adherence (75). A randomized trial evaluating the oral formulation of azacitidine (CC-486) is currently ongoing (AMADEUS trial).

As described above, IFN-α has been suggested to enhance GVL effects and has also been shown to induce apoptosis of LSCs derived from AML patients *in vitro (*91). Furthermore, Ropeg-IFN induces both apoptosis and differentiation of LSCs, indicating that Ropeg-IFN selectively targets LSCs (85). Although the immunogenicity of differentiating LSCs has not yet been fully characterized, it will be of great interest to determine whether Ropeg-IFN enhances graft-versus-leukemia stem cell effects.

Therapeutic strategies targeting TIM-3, which is expressed on LSCs, are also under development. Kikushige et al. demonstrated that administration of anti-TIM-3 antibodies suppressed the LSC fraction in immunodeficient mice transplanted with human AML cells (92). KK2845, an antibody-drug conjugate (ADC) consisting of a fully human anti-TIM-3 IgG1 antibody linked via a valine-alanine linker to a pyrrolobenzodiazepine (PBD) dimer with DNA crosslinking activity, has demonstrated potent cytotoxicity and antibody-dependent cellular cytotoxicity (ADCC) against AML cells in both *in vitro* and *in vivo* models and is currently undergoing clinical development as a novel ADC therapy for AML (93). TIM-3 is also expressed on activated and exhausted T cells, where it functions as an inhibitory immune checkpoint (94, 95). Therefore, therapeutic TIM-3 blockade may enhance antitumor immunity but could also increase the risk of autoreactive or alloreactive T-cell activation, potentially exacerbating immune-mediated toxicities including GVHD (96, 97). In contrast, antibody-drug conjugate approaches targeting TIM-3-positive AML cells may offer a more selective strategy, although careful clinical evaluation of on-target and off-tumor effects remains necessary (92, 93, 98).

#### 3.2.5 Separation of GVL and GVHD by targeting TiME

In a retrospective analysis of 409 patients with FLT3-ITD-mutated AML who relapsed after allogeneic transplantation, the complete remission (CR) rate was approximately 20% with donor lymphocyte infusion (DLI) alone and approximately 30% with sorafenib alone, whereas a CR rate exceeding 60% was observed with the combination of DLI and sorafenib. These findings suggest a synergistic interaction between graft-versus-leukemia (GVL) effects and FLT3 inhibition, and the underlying mechanisms have recently been elucidated.

In murine models, it has been demonstrated that inhibition of FLT3-ITD enhances IL-15 production by leukemia cells, thereby suppressing T-cell exhaustion and augmenting GVL effects (26, 99). Sorafenib, a multikinase inhibitor, suppresses the expression of activating transcription factor 4 (ATF4), whose expression is upregulated by FLT3-ITD signaling in FLT3-ITD-mutated leukemia cells (26). This suppression leads to derepression of interferon regulatory factor 7 (IRF7), which is normally inhibited by ATF4, resulting in enhanced phosphorylation of IRF7 and increased transcription of IL-15. Accordingly, short-term administration of sorafenib after allogeneic stem cell transplantation promotes IL-15 production by leukemia cells, which in turn increases Bcl-2 expression in donor CD8^+^ T cells, thereby enhancing their survival and limiting exhaustion. Through these mechanisms, sorafenib augments GVL activity against FLT3-ITD-mutated AML. Notably, deletion of IL-15 in leukemia cells abrogates the GVL-enhancing effect of sorafenib, indicating that post-transplant sorafenib suppresses leukemic proliferation predominantly through IL-15-mediated enhancement of GVL, rather than through direct antitumor effects. Similarly, post-transplant administration of the selective FLT3 inhibitor gilteritinib has been shown to enhance IL-15 production by FLT3-ITD-mutated leukemia cells, suppress donor CD8^+^ T-cell exhaustion, and augment cytotoxic T lymphocyte (CTL) activity against recipient leukemia, thereby promoting GVL effects (99).

In murine models, systemic administration of IL-15 after transplantation has been shown to induce a marked reduction in leukemic cells, while simultaneously provoking lethal GVHD (26). In contrast, administration of FLT3 inhibitors after allogeneic transplantation in mice does not exacerbate GVHD, and the randomized MORPHO trial, which evaluated post-transplant maintenance therapy with gilteritinib, did not significantly increase the incidence of GVHD while improving relapse-free survival in patients who were MRD-positive before or after transplantation (100). After its production, IL-15 binds to IL-15Rα and is transported to the cell surface, where it stimulates IL-2Rβ/γc-expressing cells, such as neighboring T cells, thereby activating these immune cells (101). Thus, a substantial proportion of IL-15 induced by FLT3 inhibition in leukemia cells is likely presented on the leukemia cell surface, leading to activation of adjacent T cells. In this context, IL-15 production driven by FLT3 inhibitors may alter TiME preferentially around leukemia cells, potentially avoiding the exacerbation of GVHD that is observed with systemic IL-15 administration. Moreover, because this mechanism depends on enhanced IL-15 production by leukemia cells, the immunostimulatory effect is presumed to occur only in the presence of residual leukemia.

#### 3.2.6 Enhancement of GVL effects through suppression of T cell exhaustion

As described above, donor T cells rapidly differentiate into terminally exhausted T cells and become dysfunctional in mouse models. However, we discovered that calcineurin inhibitors (CNIs), which are routinely used in transplantation, suppress terminal exhaustion of donor T cells by inhibiting the expression of TOX, a key transcription factor that regulates T cell exhaustion (102, 103). Instead, CNIs increase the population of immunocompetent donor T cells that retain alloreactivity. These CNI-induced T-cell populations contribute to the development of chronic GVHD and GVL effects induced by post-HCT administration of PD-1 inhibitors (102, 103). Furthermore, studies in mouse models demonstrated that administration of CNIs for the first 5 days after transplantation suppresses subsequent terminal exhaustion of donor T cells (104). Analysis of peripheral blood T cells on day 28 in patients underwent haploidentical HCT with post-transplant cyclophosphamide (PTCy) revealed that patients who initiated tacrolimus on day -1 exhibited an increased less exhausted T-cell populations compared with those who initiated tacrolimus on day 5. Therefore, in patients at high risk of relapse, earlier initiation of CNIs, in the context of PTCy-based transplantation, may enhance GVL effects. Notably, reducing the dose of cyclophosphamide and initiating CNIs earlier has been shown to increase GVHD risk in the context of PTCy (105). Whether optimization of cyclophosphamide dosing and CNI initiation timing can enhance GVL effects without increasing chronic GVHD remains an important question for future investigation. In addition, emerging registry studies suggest that donor-related factors, particularly younger donor age, may influence relapse risk and survival outcomes, including disease-free or event-free survival, indicating that donor selection may also contribute to optimizing the GVL/GVHD balance.

## 4 Summary and future directions

**Figure 1:**
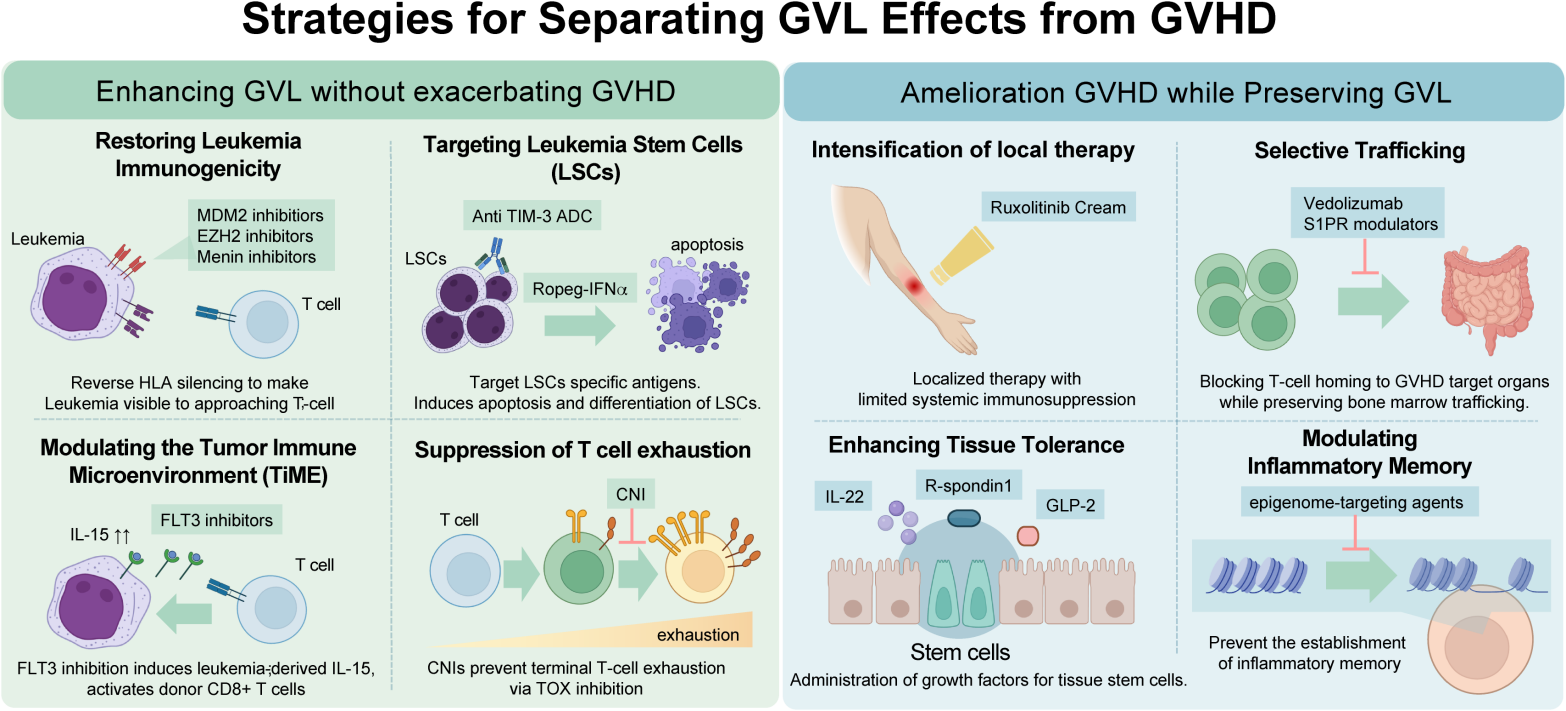
Strategies for separating graft-versus-leukemia effects from graft-versus-host disease. This schematic summarizes representative approaches to dissociate graft-versus-leukemia (GVL) effects from graft-versus-host disease (GVHD). The left panel highlights strategies to enhance GVL without exacerbating GVHD. These include restoration of leukemia immunogenicity through reversal of HLA silencing using agents such as MDM2 inhibitors, EZH2 inhibitors, and menin inhibitors; targeting of leukemia stem cells (LSCs) using anti-TIM-3 antibody-drug conjugates and ropeginterferon-α (Ropeg-IFNα), which may induce apoptosis and differentiation of LSCs; modulation of the tumor immune microenvironment (TiME), such as FLT3 inhibitor–induced leukemia-derived IL-15 production that activates donor CD8^+^ T cells; and suppression of terminal T-cell exhaustion by calcineurin inhibitors (CNIs) through inhibition of TOX. The right panel summarizes strategies to ameliorate GVHD while preserving GVL. These include intensification of local therapy, such as topical ruxolitinib for cutaneous GVHD; selective inhibition of donor T-cell trafficking to GVHD target organs using vedolizumab or sphingosine-1-phosphate receptor (S1PR) modulators while preserving bone marrow trafficking; enhancement of tissue tolerance through administration of tissue stem cell–supporting factors, including IL-22, R-spondin1, and GLP-2; and modulation of inflammatory memory using epigenome-targeting agents to prevent persistent epithelial sensitization after inflammation. Together, these approaches illustrate complementary strategies aimed at maximizing anti-leukemia immunity while minimizing tissue injury after allogeneic hematopoietic cell transplantation. The figure was created using BioRender.com.

Recent advances in understanding the pathophysiology of GVHD and the mechanisms underlying leukemia relapse are expected to make the long-considered difficult goal of separating GVHD from GVL effects achievable in the near future (**Figure 1**). Accumulating insights into HLA loss, impaired antigen presentation, donor T-cell exhaustion, leukemia stem cell persistence, and the tumor immune microenvironment have created new opportunities to enhance GVL effects while limiting GVHD. In addition to the strategies discussed above, emerging cellular approaches may offer complementary routes to further dissociate GVL from GVHD, particularly given the overlap of alloantigens, including minor histocompatibility antigens, between leukemia cells and normal recipient tissues. γδ T cells can enhance antitumor immunity while mitigating GVHD (106, 107). Regulatory T-cell-based approaches, including CAR-Treg platforms and minor histocompatibility antigen-specific regulatory T cells, can selectively suppress pathogenic alloresponses while preserving beneficial immunity (108, 109). Although these approaches remain at an early stage of development, they may expand future options for precise immune modulation after allo-HCT. Continued integration of molecular profiling of leukemia cells and immune cells, immunological insights, and translational research will be essential to further refine strategies that dissociate GVL from GVHD in patients undergoing allo-HCT and to translate these advances into clinically effective precision therapies.
